# NK cells contribute to reovirus-induced IFN responses and loss of tolerance to dietary antigen

**DOI:** 10.1172/jci.insight.159823

**Published:** 2022-08-22

**Authors:** Pamela H. Brigleb, Elaine Kouame, Kay L. Fiske, Gwen M. Taylor, Kelly Urbanek, Luzmariel Medina Sanchez, Reinhard Hinterleitner, Bana Jabri, Terence S. Dermody

**Affiliations:** 1 Department of Microbiology and Molecular Genetics, University of Pittsburgh School of Medicine, Pittsburgh, Pennsylvania, USA.; 2 Institute of Infection, Inflammation, and Immunity, UPMC Children’s Hospital of Pittsburgh, Pittsburgh, Pennsylvania, USA.; 3 Committee on Immunology, University of Chicago, Chicago, Illinois, USA.; 4 Department of Pediatrics and; 5 Department of Immunology, University of Pittsburgh School of Medicine, Pittsburgh, Pennsylvania, USA.; 6 Department of Medicine, University of Chicago, Chicago, Illinois, USA.

**Keywords:** Immunology, Virology, Innate immunity, NK cells, Th1 response

## Abstract

Celiac disease is an immune-mediated intestinal disorder that results from loss of oral tolerance (LOT) to dietary gluten. Reovirus elicits inflammatory Th1 cells and suppresses Treg responses to dietary antigen in a strain-dependent manner. Strain type 1 Lang (T1L) breaks oral tolerance, while strain type 3 Dearing reassortant virus (T3D-RV) does not. We discovered that intestinal infection by T1L in mice leads to the recruitment and activation of NK cells in mesenteric lymph nodes (MLNs) in a type I IFN–dependent manner. Once activated following infection, NK cells produce type II IFN and contribute to IFN-stimulated gene expression in the MLNs, which in turn induces inflammatory DC and T cell responses. Immune depletion of NK cells impairs T1L-induced LOT to newly introduced food antigen. These studies indicate that NK cells modulate the response to dietary antigen in the presence of a viral infection.

## Graphical abstract

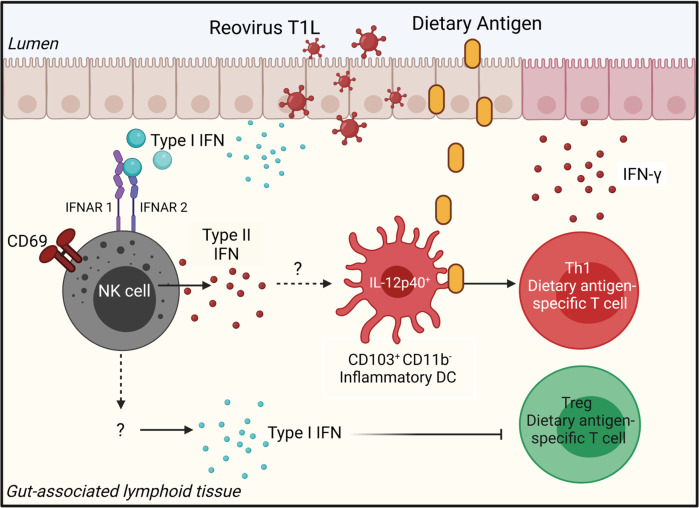

## Introduction

Celiac disease (CeD) is an autoimmune disorder characterized by an inflammatory Th1 response to dietary gluten (1–4). There are currently limited treatment options and no cure exists. Approximately 1 in 133 persons in the United States has CeD, and prevalence has increased over the past decade (1, 5–7). Individuals with human leukocyte antigen DQ2 or DQ8 alleles are genetically predisposed to CeD, with approximately 90% of individuals diagnosed with CeD having 1 or both alleles (2, 3, 8–10). While approximately 40% of the US population has these genetic determinants, only approximately 1% of the population has CeD (4, 5). This discrepancy indicates that additional environmental or genetic factors contribute to disease onset. CeD development has been linked to several viruses, including adenovirus, norovirus, and mammalian orthoreovirus (reovirus) (11–16). However, the mechanisms by which viruses trigger CeD are unknown.

Reovirus is an enteric, nonenveloped virus with a segmented dsRNA genome (17). Most individuals are first infected with reovirus in childhood and experience recurrent infections throughout life. Although reovirus infections are generally asymptomatic (18), persons with CeD have higher reovirus Ab titers than unaffected individuals, suggesting a link between reovirus and CeD (14). We found that reovirus infection in mice triggers loss of oral tolerance (LOT) to OVA as a model food antigen in a virus strain-dependent manner. Reovirus strain type 1 Lang (T1L) elicits a proinflammatory response mediated by type I IFN and IFN regulatory factor-1 (IRF1) that culminates in induction of OVA-specific Th1 T cells and suppression of Tregs (14). However, a strain type 3 Dearing reassortant virus (T3D-RV) that infects the intestine comparably to T1L induces a less robust inflammatory response, including less type I IFN production and not blocking oral tolerance (OT) to OVA (14, 19). This model of virus-induced LOT to dietary antigen provides a unique opportunity to investigate mechanisms of virus-induced CeD, which may illuminate new therapeutic targets.

The intestine is a mucosal tissue network that incorporates multiple defense mechanisms to protect against enteric pathogens. Reovirus can traverse the intestinal epithelial barrier after peroral (PO) inoculation and replicate in gut-associated lymphoid tissue (GALT) (14, 20). In the context of LOT, the inflammatory anti-OVA response induced by T1L is characterized by Th1 CD4^+^ T cells that are primed by migratory CD103^+^CD11b^–^ intestinal DCs (21–23). However, the mechanisms by which T1L induces this inflammatory immune environment are unknown. One possibility is that reovirus replication elicits strain-specific innate immune responses in regions of GALT required for tolerance induction, including Peyer’s patches (PPs) and mesenteric lymph nodes (MLNs), producing inflammatory responses required for LOT. NK cells are innate immune cells that function in the detection and killing of virus-infected cells. These cells contribute to inflammation and tissue injury caused by other viruses at mucosal barriers (24–26) and can respond to or secrete cytokines such as type II IFN (27–29). Proinflammatory cytokines including type II IFN can activate inflammatory DCs and prime subsequent Th1 responses (28, 30–32) as well as mediate intestinal tissue damage associated with CeD (1, 14, 33).

Innate immune responses elicited by reovirus intestinal infection, including the innate immune cells recruited and their functions in antiviral defense and LOT, are not well understood. This knowledge gap is particularly important since innate immune responses influence adaptive immune responses, including DC priming and T cell activation, required for OT. Here, we report that T1L intestinal infection elicits NK cell recruitment and NK-mediated inflammatory type II IFN production dependent on type I IFN. We discovered that NK cells elicit CD103^+^CD11b^–^ intestinal DC migration and activation and contribute to both inflammatory Th1 responses and suppression of Treg expansion specific for dietary antigen during T1L infection. Our results define cellular innate immune responses induced by reovirus in the intestine and identify NK cells as a potential therapeutic target for virus-induced CeD.

## Results

### T1L intestinal infection results in an increase of NK cells in GALT.

**Figure 1:**
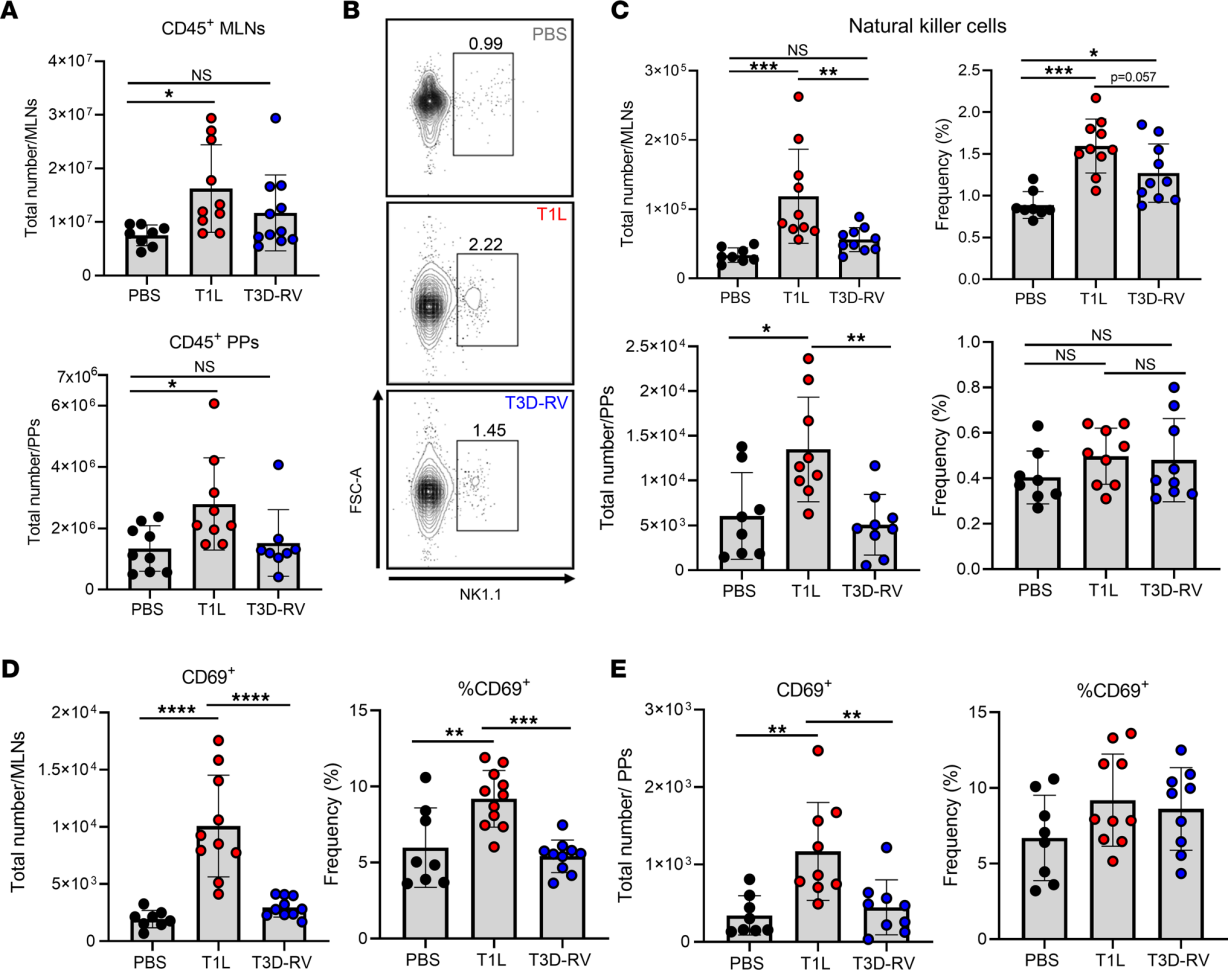
Reovirus strains differ in NK cell infiltration and general activation in GALT early following inoculation. WT mice were PO inoculated with 1 × 10^8^ PFU of T1L or T3D-RV or PBS as a control. At 2 dpi, MLNs and PPs were resected and processed for flow cytometry and determination of viral titer in a 3 cm section of ileal tissue to ensure titer-matched samples. Single-cell suspensions were stained with a comprehensive Ab panel and analyzed by flow cytometry (*n* = 6–11). (**A**) Total immune cells (CD 45^+^) in MLNs or PPs following inoculation. (**B**) Dot plots of representative samples of NK cells (CD45^+^ TCRβ^–^ NK1.1^+^) in MLNs at 2 dpi. (**C**) Total cell count and percent of NK cells (CD45^+^ TCRβ^–^ NK1.1^+^) in MLNs and PPs. (**D**) Total number and percent of activated CD69-expressing NK cells in MLNs. (**E**) Total number and percent of activated CD69-expressing NK cells in PPs. Results are presented as mean values. Data are shown as mean ± SEM. Statistical significance was calculated using 1-way ANOVA with Tukey’s multiple comparisons test in **A** and **C**–**E**. *P < 0.05; **P < 0.01; ***P < 0.001; ****P < 0.0001.

Reovirus strains T1L and T3D-RV replicate comparably in cell culture and infect the murine intestine (14, 19). However, T1L induces a greater overall inflammatory response as determined by a significant increase in CD45^+^ immune cell infiltration in both MLNs and PPs (Figure 1A) compared with PBS (mock) and T3D-RV. This difference in total immune cell infiltration to GALT displayed by T1L and T3D-RV early in infection suggests that there are strain-specific differences in the induction of innate immune responses that may influence adaptive immune responses associated with LOT.

As NK cells mediate antiviral activities and influence both antigen presentation and T cell responses (28, 29, 31, 34), we focused on functions of NK cells during reovirus infection. Using flow cytometry, we discovered that T1L infection is associated with a significant increase in the number and percentage of NK cells (CD45^+^TCRβ^–^NK1.1^+^) in MLNs and more modestly in PPs compared with T3D-RV at 2 days post infection (dpi) (Figure 1, B and C). Increases in NK cells were observed at 1 dpi with more robust responses at 2 dpi (Supplemental Figure 1, A and B; supplemental material available online with this article; https://doi.org/10.1172/jci.insight.159823DS1). We assessed NK cell activation by CD69 expression, which indicates cytolytic activity (35–39). NK cells expressed significantly higher levels of CD69 in MLNs during T1L infection than during T3D-RV or mock infection (Figure 1D) and more modestly in PPs (Figure 1E). These findings were more predominant at 2 dpi (Supplemental Figure 1B). We also examined NKT cell populations in the MLNs following infection and found that the prevalence of these cells in the MLNs was low compared with NK cells. No differences in the frequency or total number of NKT cells were observed between uninfected and T1L-infected mice (Supplemental Figure 1, C and D). These data suggest that T1L infection elicits recruitment and activation of NK cells in MLNs, which may contribute to the inflammatory immune responses that break OT.

### NK cells produce type II IFN during T1L infection to mediate inflammatory responses in GALT.

**Figure 2:**
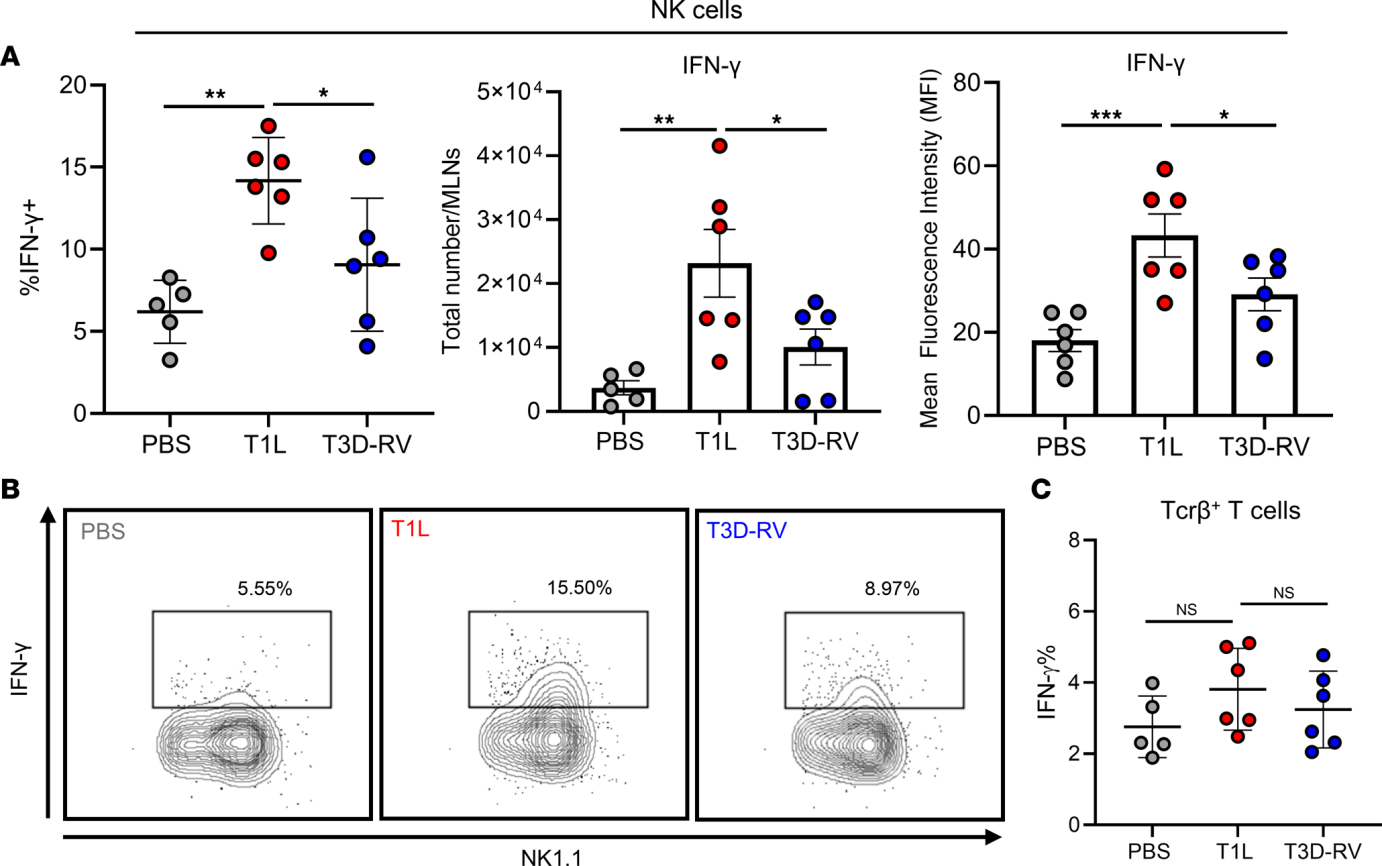
Reovirus strain T1L induces more type II IFN production in NK cells. WT mice were PO inoculated with 1 × 10^8^ PFU of T1L or T3D-RV or PBS as a control. At 2 dpi, MLNs and PPs were resected and processed for flow cytometry and determination of viral titer in a 3 cm section of ileal tissue to ensure titer-matched samples. MLN cell suspensions (5 × 10^6^ cells) were stimulated with PMA and ionomycin at 37°C for 4 hours in the presence of brefeldin A, and cells were assessed for type II IFN production by intracellular cytokine staining (*n* = 5–6). (**A**) Percentage, total number, and MFI of NK cells expressing type II IFN. (**B**) Contour plot of intracellular type II IFN expression by a representative sample of NK cells (CD45^+^ TCRβ^–^ NK1.1^+^). (**C**) Percentage of T cells (CD45^+^ Tcrβ^+^) expressing type II IFN. Results are presented as mean values. Data are shown as mean ± SEM. Statistical significance was calculated using 1-way ANOVA with Tukey’s multiple comparisons test in **A** and **C**. *P < 0.05; **P < 0.01; ***P < 0.001.

Since the most striking differences in NK cells and NK cell activation were observed in MLNs, which is the site of OT induction, we characterized NK cell responses in MLNs following reovirus infection. T1L-mediated inflammatory Th1 responses to dietary antigen are dependent on IRF1, which can be activated by type II IFN signaling. Therefore, we identified immune cells that increase type II IFN production following reovirus infection in a strain-dependent manner. Mice were PO inoculated with T1L, T3D-RV, or PBS (mock), and single-cell suspensions from MLNs were stimulated and stained for intracellular type II IFN at 2 dpi. Type II IFN production in NK cells was significantly increased following infection with T1L compared with T3D-RV and mock (Figure 2, A and B). Type II IFN expression in T cell populations (TCRβ^+^) was not altered following infection with either T1L or T3D-RV (Figure 2C), suggesting that T cells do not contribute to increased type II IFN expression early in infection. These data indicate that NK cells respond to type I IFN during T1L infection, resulting in production of type II IFN and inflammation in GALT.

### Increases in NK cells following infection are due to enhanced recruitment.

**Figure 3:**
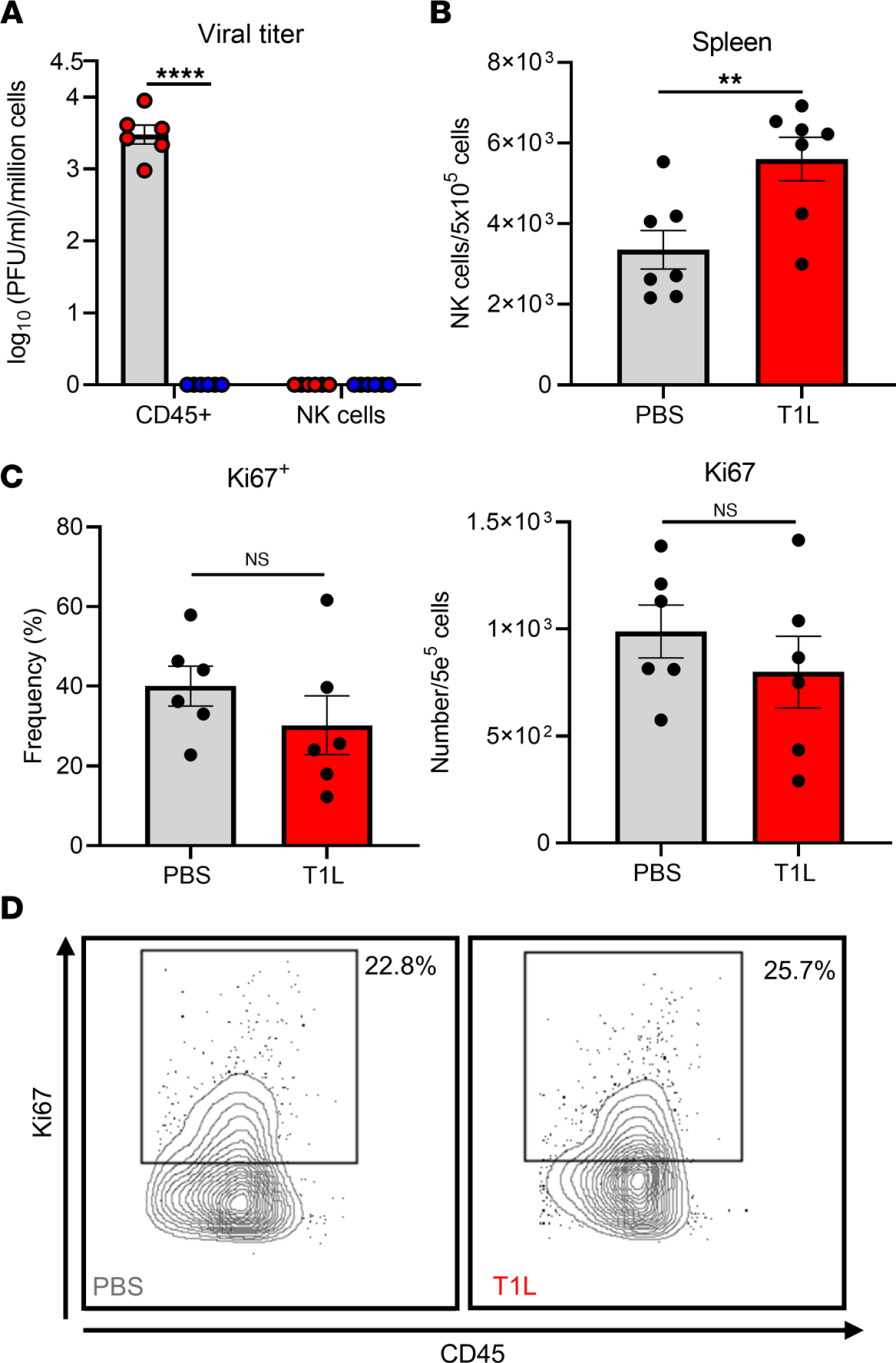
NK cells are not susceptible to reovirus infection and are recruited from the bone marrow and do not increase proliferation at site of infection. (**A**) WT mice were PO inoculated with 1 × 10^8^ PFU of T1L or T3D-RV or PBS as a control. At 2 dpi, single-cell suspensions from MLNs were sorted by flow cytometry to obtain live, CD45^+^ immune cells or NK1.1^+^Tcrβ^–^ NK cells and processed for viral titer by plaque assay (*n* = 6). (**B**) WT mice were PO inoculated with 1 × 10^8^ PFU of T1L or PBS as a control. At 2 dpi, the spleen was resected and processed for flow cytometry to determine the number of NK cells in the spleen (*n* = 7). (**C**) WT mice were PO inoculated with 1 × 10^8^ PFU of T1L or PBS as a control. At 2 dpi, MLNs were resected and processed for flow cytometry and were assessed for ki67 marker of proliferation by intracellular cytokine staining (*n* = 6). (**D**) Representative contour plots of ki67 staining in NK cells (CD45^+^ Tcrβ^–^ NK1.1^+^ DX5^+^ ki67^+^). Results are presented as mean values. Data are shown as mean ± SEM. Statistical significance was calculated using 1-way ANOVA with Tukey’s multiple comparisons test in **A** or using Student’s *t* test in **B** and **C**. **P < 0.01; ****P < 0.0001.

A possible explanation of why an increase in NK cells was observed during T1L infection is that NK cells are being infected, thereby affecting NK cell infiltration and cytokine production. We tested whether NK cells are infected by reovirus in a strain-dependent manner, resulting in proinflammatory cytokine production and increased NK cell numbers in GALT during T1L infection. We sorted either total CD45^+^ cells or NK cells (CD45^+^TCRβ^–^NK1.1^+^) from MLNs at 2 days following PO inoculation of T1L, T3D-RV, or PBS and determined viral titers in cell suspensions by plaque assay. We detected infectious T1L but not T3D-RV in total CD45^+^ immune cells (Figure 3A). However, we did not detect infectious virus in the NK cell population (Figure 3A), indicating that T1L does not infect NK cells.

NK cells are derived from the lymphoid progenitor lineage and mature in the bone marrow prior to release into the circulation and migration to distant sites including lymph nodes (40). The significant increase in the total number of NK cells in the MLNs following T1L infection compared with mock-infected controls could be attributable to NK cell recruitment, proliferation, or both. To distinguish between these possibilities, we quantified NK cells in the spleen following infection as a surrogate of NK cells in the blood (recruitment) and assessed proliferation of NK cells in the MLNs by staining for the proliferation marker ki67 (expansion). We found that there was a significant increase in the number of NK cells in the spleen following infection (Figure 3B), indicating that there is enhanced NK cell recruitment during infection. In contrast, there were no differences in the percentage and total number of NK cells expressing ki67 in T1L-infected animals and mock controls (Figure 3, C and D). These data suggest that the significant increase in NK cells in the MLNs is attributable to enhanced recruitment and not expansion of NK cells.

### NK cell-mediated inflammatory responses are specific to type II IFN.

Type II IFN can mediate inflammatory DC activation and contribute to type I IFN and IRF1 responses that are essential for T1L-induced LOT. We demonstrated that NK cells significantly increase type II IFN production following T1L infection (Figure 2, B and C). However, there are other potential mechanisms of NK cell-mediated DC activation. GM-CSF produced by NK cells can activate cDC1 to mediate Th1 CD4^+^ T cell inflammatory responses in the lymph nodes (41). To determine whether NK cells also produce GM-CSF that may contribute to DC activation, we PO inoculated mice with T1L or PBS as a mock control and stained NK cells for intracellular GM-CSF expression. There was no increase in the percentage of total immune cells (CD45^+^) that express intracellular GM-CSF during T1L infection (Supplemental Figure 2A). There also was no difference in the number of GM-CSF–expressing NK cells and a significant decrease in the percentage of NK cells that express GM-CSF (Supplemental Figure 2B). These data suggest that NK cells do not increase GM-CSF expression during T1L infection and, therefore, GM-CSF likely does not contribute to inflammatory DC activation.

### Type I IFN restricts T1L replication and modulates NK cell infiltration and activation in GALT.

**Figure 4:**
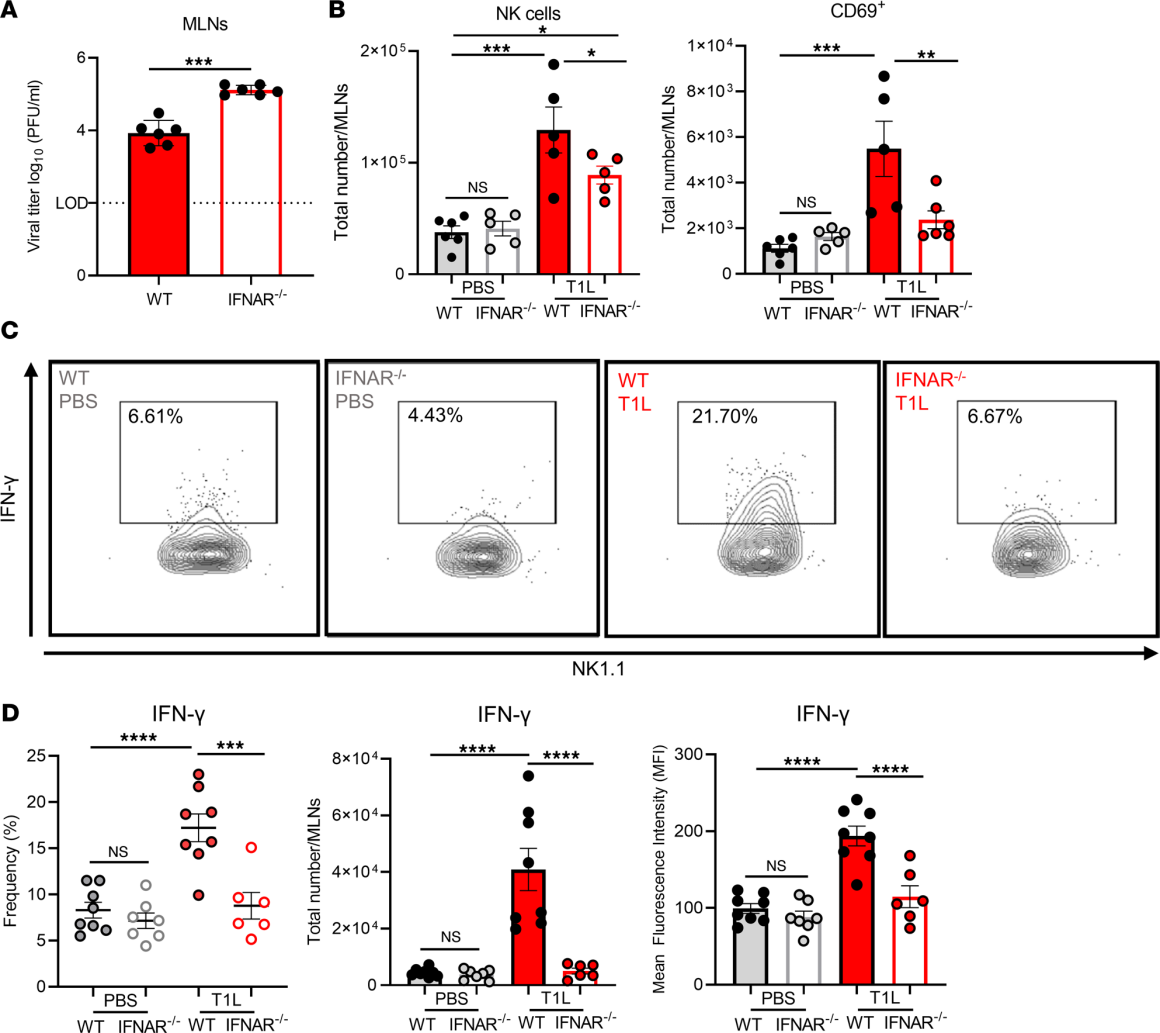
Type I IFN modulates NK cell recruitment, activation, and type II IFN responses in MLNs. WT or *IFNAR^–/–^* mice were PO inoculated with 1 × 10^8^ PFU of T1L or PBS as a control. At 2 dpi, MLNs were resected and processed for flow cytometry (*n* = 5–6). (**A**) Viral titers in MLNs were determined by plaque assay at 2 dpi (*n* = 6) (**B**) Number of NK cells (CD45^+^ TCRβ^–^ NK1.1^+^) and number of NK cells expressing activation marker CD69 (CD45^+^ TCRβ^–^ NK1.1^+^ CD69^+^) in MLNs. (**C** and **D**) MLN cell suspensions (5 × 10^6^ cells) were stimulated with PMA and ionomycin at 37°C for 4 hours, and cells were assessed for IFN-γ production by intracellular cytokine staining (*n* = 6–8). (**C**) Number, percentage, and MFI of NK cells (CD45^+^ TCRβ^–^ NK1.1^+^) that express intracellular IFN-γ. (**D**) Contour plot of intracellular IFN-γ expression by a representative sample of NK cells (CD45^+^ TCRβ^–^ NK1.1^+^). Results are presented as mean values. Data are shown as mean ± SEM. Statistical significance was calculated using Student’s *t* test in **A** and 1-way ANOVA with Tukey’s multiple comparisons test in **B**–**D**. *P < 0.05; **P < 0.01; ***P < 0.001; ****P < 0.0001.

Type I IFN is required for blockade of Treg differentiation in response to dietary antigen during T1L infection (14). Since NK cells upregulate type II IFN in response to T1L infection (Figure 2), we tested whether total immune cell recruitment and NK cell infiltration is dependent on type I IFN. WT or *IFNAR*^–/–^ mice were PO inoculated with T1L or PBS (mock), and viral titers and immune cell populations were assessed. T1L produced significantly higher loads in both intestinal tissue and GALT in *IFNAR*^–/–^ mice at 2 dpi compared with loads produced in WT mice, indicating that type I IFN restricts T1L replication (Figure 4A), consistent with previous findings (14, 42). Since T1L infection results in increased NK cell recruitment and activation in GALT (Figures 1 and 3), we determined whether these effects occur in *IFNAR*^–/–^ mice. The total number of NK cells were substantially decreased following T1L infection of *IFNAR*^–/–^ mice compared with WT mice (Figure 4B), and the number of NK cells expressing cytolytic activation marker CD69 were significantly reduced (Figure 4B). We also observed a significant reduction in the number and percentage of IFN-γ–producing NK cells in *IFNAR*^–/–^ mice compared with WT mice following T1L infection (Figure 4, C and D). NK cell-produced type II IFN also can be mediated by IL-12 signaling. To determine whether IL-12 is required for NK cell type II IFN production during T1L infection, we immunodepleted IL-12 and found that diminished IL-12 did not reduce the capacity of NK cells to produce type II IFN during T1L infection (Supplemental Figure 2C). These data indicate that NK cell infiltration, activation, and type II IFN production are mediated by type I IFN responses during T1L infection and suggest a function for NK cells in reovirus-triggered LOT.

### NK cells are dispensable for viral clearance but mediate T1L-induced inflammatory responses in the MLNs.

**Figure 5:**
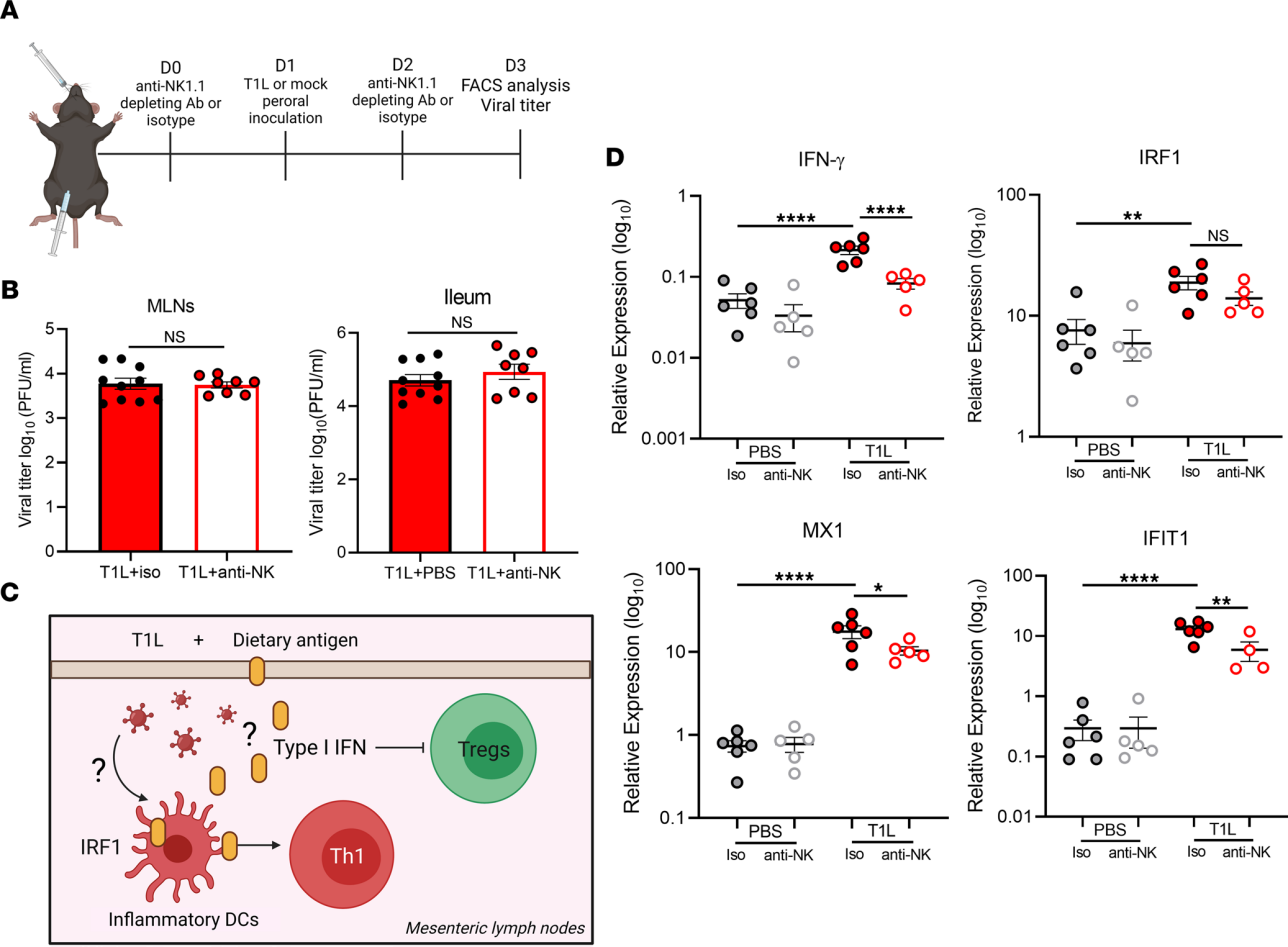
NK cells are dispensable for viral clearance but contribute to IFN-mediated inflammatory responses that are associated with loss of tolerance to dietary antigen. WT mice were i.p. injected with either isotype control IgG2a Ab or anti-NK1.1 Ab (PK136) 1 day prior to and 1 day following PO inoculation with 1 × 10**^8^** PFU of T1L or PBS as a control. At 2 dpi, MLNs were resected and processed for flow cytometry (*n* = 8–10). (**A**) Design of NK cell depletion studies. (**B**) Viral titers in MLNs and a 3 cm section of the ileum determined by plaque assay at 2 dpi. (**C**) Model of immune pathways involved in T1L-induced LOT made with Biorender. (**D**) Type I and type II IFN–dependent gene expression compared with GAPDH in MLNs determined by qPCR at 2 dpi. Results are presented as mean values. Data are shown as mean ± SEM. Statistical significance was calculated using Student’s *t* test in **B** and 1-way ANOVA with Tukey’s multiple comparisons test in **D**. *P < 0.05; **P < 0.01; ****P < 0.0001.

To determine whether NK cells contribute to antiviral inflammation, we depleted NK cells in WT mice using an anti-NK1.1 immune-depleting Ab (Figure 5A, and Supplemental Figure 3A). Anti-NK1.1–treated mice and those treated with an isotype control Ab were PO inoculated with T1L or PBS (mock), and viral titers in intestinal and GALT were determined at 2 dpi. NK cell depletion did not lead to statistically significant alterations in viral titers in the MLNs or ileum (Figure 5B), suggesting that NK cells do not contribute to viral clearance early in infection.

Type I IFN and IRF1 are required for T1L-induced LOT (Figure 5C) (14). However, the immune cells that regulate these responses are unknown. To determine whether NK cells contribute to LOT following T1L infection, we depleted NK cells using the anti-NK1.1 Ab and assessed effects on induction of innate and adaptive immune responses associated with reovirus-induced LOT (Figure 5A). At 2 dpi, NK cell depletion significantly decreased expression of type I IFN–stimulated genes (ISGs), including IFIT1 and MX1, and significantly decreased expression of type II IFN and a trend toward decreased expression of IRF1 in the MLNs (Figure 5D). These findings suggest that NK cells contribute to inflammatory responses known to mediate reovirus-induced LOT.

### NK cells mediate CD103^+^CD11b^–^ DC responses in MLNs during T1L infection.

Tolerogenic DCs present dietary antigen to T cells in the MLNs (43, 44). CD103^+^CD11b^–^ migratory DCs have the highest tolerogenic potential (14, 21, 45). To characterize DC-specific responses in the MLNs, we quantified the migratory DC populations during T1L infection with and without NK cell depletion (Supplemental Figure 3B). There was a significant influx of CD103^+^CD11b^–^ migratory DCs to the MLNs following T1L infection compared with mock-infected controls (Supplemental Figure 3C). NK cell depletion quenched this effect, as the number of CD103^+^CD11b^–^ migratory DCs in MLNs of reovirus-infected mice following NK cell depletion was comparable to those in mock-infected controls (Supplemental Figure 3C).

**Figure 6:**
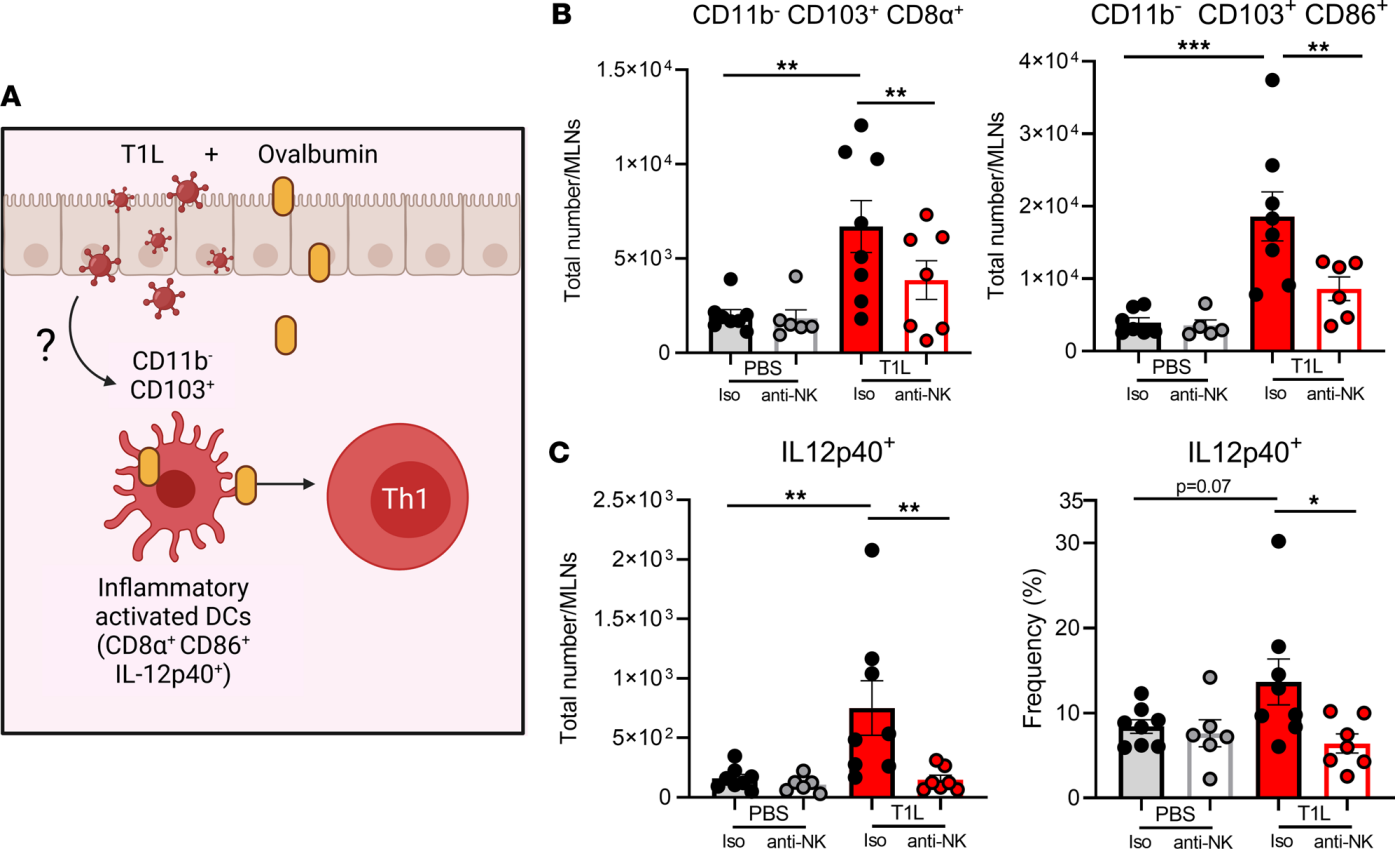
NK cells contribute to CD103^+^CD11b^–^ DC responses and inflammatory activation in the MLNs. WT mice were i.p. injected with either isotype control IgG2a Ab or anti-NK1.1 Ab (PK136) 1 day prior to and 1 day following PO inoculation with 1 × 10**^8^** PFU of T1L or PBS as a control. At 2 dpi, MLNs were resected and processed for flow cytometry. Single-cell suspensions were incubated with brefeldin A in the presence of Golgi Plug at 37°C for 6 hours (*n* = 6–8). (**A**) Model of inflammatory DC activation important in LOT to dietary antigen made with Biorender. (**B**) Total number of CD103^+^CD11b^–^ DCs that express CD8α or CD86. (**C**) Percentage and total number of migratory tolerogenic DCs that express intracellular IL-12p40. Results are presented as mean values. Data are shown as mean ± SEM. Statistical significance was calculated using a 1-way ANOVA with Tukey’s multiple comparisons test in **B** and **C**. *P < 0.05; **P < 0.01; ***P < 0.001.

During T1L-induced LOT, CD103^+^CD11b^–^ migratory DCs become activated and induce inflammation, triggering Th1 responses to dietary antigen (14). To determine whether NK cells influence DC activation in GALT, we quantified CD103^+^CD11b^–^ DCs that express surface CD86, CD8α, and intracellular IL-12p40 (14). CD86 is a type I IFN–induced inflammatory activation marker upregulated by DCs that stimulate Th1 responses (46, 47). CD103^+^CD11b^–^ CD8α^+^ DCs take up orally administered OVA more efficiently than do other DC subsets independent of viral infection (14) (Figure 6A). IL-12 production by these DCs elicits Th1 responses (14, 15). NK-cell depletion significantly reduced the number of CD103^+^CD11b^–^ DCs expressing CD86 or CD8α following T1L infection (Figure 6B), but the frequency of these populations remained unchanged (Supplemental Figure 3D). The percentage and total number of CD103^+^CD11b^–^CD8α^+^ DCs expressing intracellular IL-12p40 also were significantly diminished (Figure 6C). Collectively, these data suggest that NK cells modulate CD103^+^CD11b^–^ DC responses in the MLNs following T1L infection, which may contribute to T1L-induced LOT.

### NK cells contribute to host antiviral responses.

**Figure 7:**
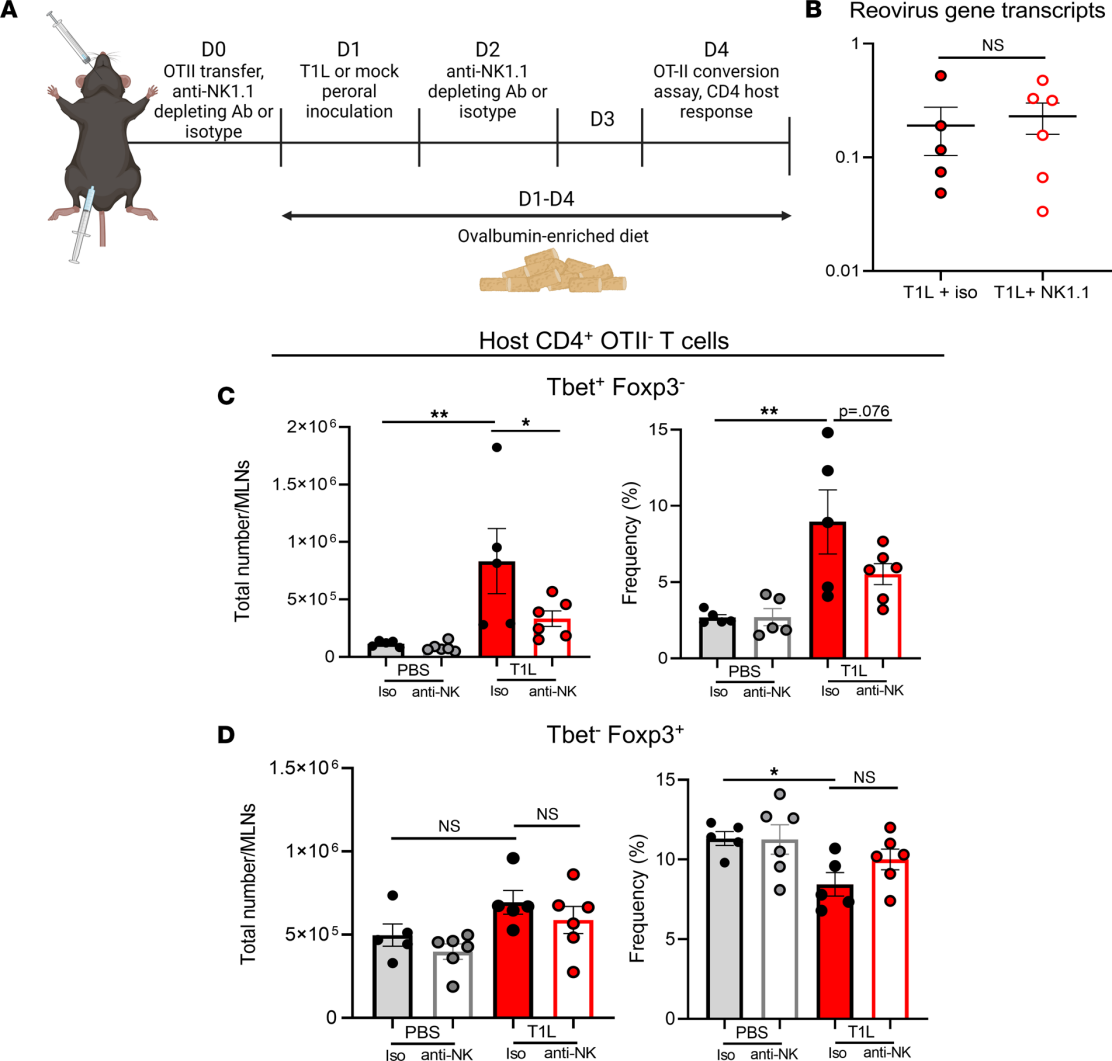
NK cells contribute to antiviral host CD4 Th1 T cell responses. WT mice were injected i.p. with either isotype control IgG2a Ab or anti-NK1.1 Ab (PK136) 1 day prior to and 1 day following PO inoculation with 1 × 10**^8^** PFU of T1L or PBS as a control. OT-II T cells were transferred from CD45.1^+^ WT mice to CD45.2^+^ WT mice 1 d prior to reovirus inoculation. At 3 dpi, MLNs were resected and processed for flow cytometry (*n* = 5–6). (**A**) Schematic of experimental procedure made with Biorender. (**B**) Reovirus gene transcripts (S4 gene) in MLNs determined by qPCR at 3 dpi. (**C**) Total number and frequency of host Th1 (Tbet^+^, Foxp3^–^) CD4^+^ T cells (CD45.1^–^ CD4^+^). (**D**) Total number and frequency of Treg (Tbet^–^, Foxp3^+^) host CD4^+^ T cells (CD45.1^–^ CD4^+^). Results are presented as mean values. Data are shown as mean ± SEM. Statistical significance was calculated using Student’s *t* test in **B** and 1-way ANOVA with Tukey’s multiple comparisons test in **C** and **D**. *P < 0.05; **P < 0.01.

To determine whether NK cells contribute to T cell responses following T1L infection, we depleted these cells using anti-NK1.1 Ab and assessed tolerance loss at 3 dpi using the T cell transfer, OVA oral antigen tolerance model (Figure 7A). Similar to the results obtained at 2 dpi, NK cell depletion did not affect viral gene transcript levels in the MLNs (Figure 7B). Infection of the murine intestine by T1L results in priming and activation of Th1 CD4^+^ T cell responses (14), but mediators of these T cell responses are unknown. We found that NK cell-depletion prior to T1L infection did not alter Treg responses (Figure 7C) but did diminish Th1 CD4^+^ T cell responses (Figure 7D). Therefore, we thought it possible that NK cells might contribute to the inflammatory responses that mediate reovirus-induced LOT to dietary antigen.

### NK cells contribute to T1L-induced LOT to dietary antigen.

**Figure 8:**
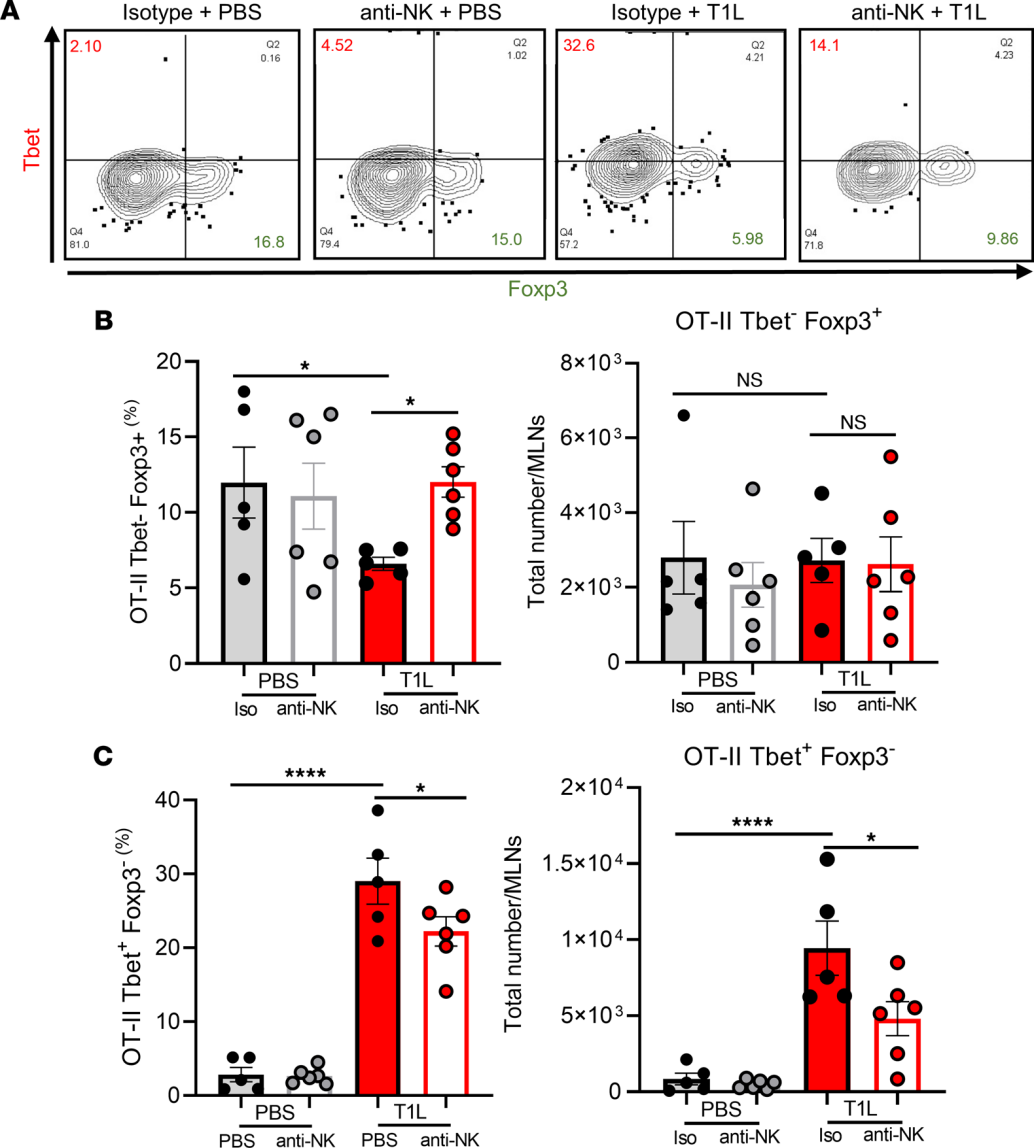
NK cells contribute to T1L-induced T cell priming responses associated with LOT to dietary antigen early following inoculation. WT mice i.p. injected with either isotype control IgG2a Ab or anti-NK1.1 Ab (PK136) 1 day prior to and 1 day following PO inoculation with 1 × 10**^8^** PFU of T1L or PBS as a control. OT-II T cells were transferred from CD45.1^+^ WT mice to CD45.2^+^ WT mice 1 day prior to reovirus inoculation. At 3 dpi, MLNs were resected and processed for flow cytometry (*n* = 5–6). (**A**) Contour plot of OT-II T cell expression of transcription factors Tbet (y-axis), indicating a Th1 phenotype, and Foxp3 (x-axis), indicating a Treg phenotype. (**B**) Total number and frequency of Treg (Tbet^–^, Foxp3^+^) OT-II CD4^+^ T cells (CD45.1^+^ CD4^+^). (**C**) Total number and frequency of Th1 (Tbet^+^, Foxp3^–^) OT-II CD4^+^ T cells (CD45.1^+^ CD4^+^). Results are presented as mean values. Data are shown as mean ± SEM. Statistical significance was calculated using 1-way ANOVA with Tukey’s multiple comparisons test in **B** and **C**. *P < 0.05; ****P < 0.0001.

**Figure 9:**
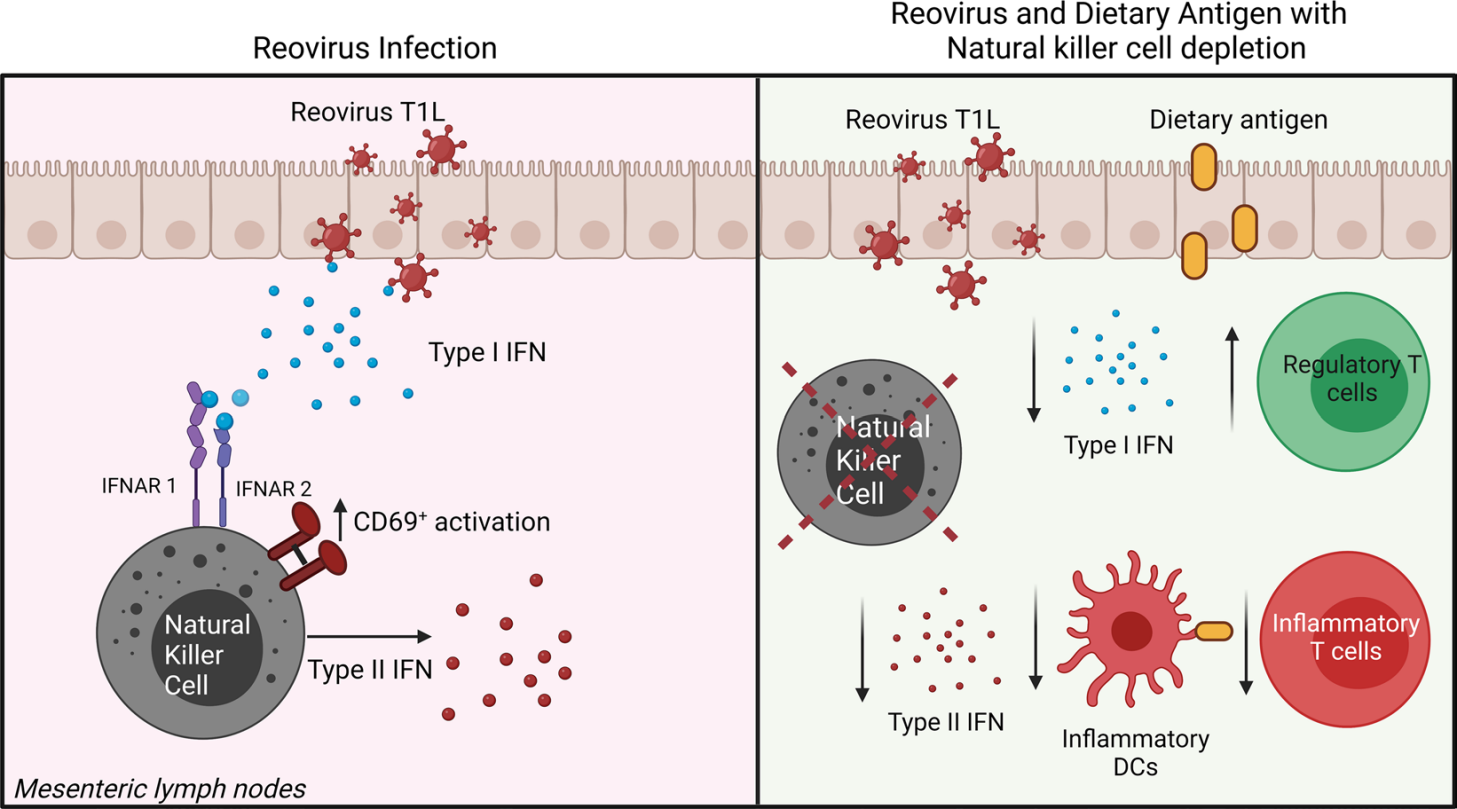
Model of NK cells as innate immune effectors during reovirus intestinal infection and effects of NK cell depletion on reovirus-induced LOT. Figure made using Biorender.

LOT to dietary OVA is characterized by induction of Tbet^+^ Foxp3^–^ OVA-specific Th1 CD4^+^ T cells and suppression of Tbet^–^ Foxp3^+^ OVA-specific Treg cells (14, 15). Intestinal T1L infection at the time of initial OVA administration leads to enhanced OVA-specific Th1 CD4^+^ T cell responses and decreased OVA-specific Treg responses (14). Tbet^+^ Foxp3^–^ OVA-specific CD4^+^ T cells are the main type II IFN–producing T cells that drive inflammatory responses to dietary antigen (14, 15). NK cell depletion significantly restored the percentage of OVA-specific Tbet^–^ Foxp3^+^ Treg cells (Figure 8, A and B), although total Treg cell numbers were unchanged (Figure 8B). Furthermore, NK cell depletion significantly blunted the OVA-specific Tbet^+^ Foxp3^–^ Th1 response following T1L infection (Figure 8, A and C). Collectively, these data demonstrate that NK cells contribute to both the priming of T1L-mediated inflammatory Th1 responses and Treg suppression to dietary antigen early following infection, as shown schematically in Figure 9, and suggest that NK cells are a key determinant of virus-induced CeD pathogenesis.

## Discussion

HLA DQ2 and DQ8 alleles are genetic risk factors for CeD, but these alleles do not explain the discrepancy between the percentage of those who have the genetic risk and those who develop the disease (4, 5, 7). Reovirus can trigger LOT to newly introduced food antigen in a viral strain-dependent manner (14). We investigated immune responses evoked by reovirus strain T1L, which blocks development of oral immunological tolerance, and identified pathways that are required for virus-induced tolerance loss. We discovered that intestinal T1L infection elicits enhanced recruitment and activation of NK cells in GALT (Figures 1–4), which contribute to intestinal DC and dietary antigen-specific T cell responses (Figures 5–9). These data provide insights into pathways that may lead to virus-induced CeD.

Functions of NK cells during reovirus infection are not well characterized. However, ingenuity pathway analysis (48, 49) of previously published RNA sequencing data (14) indicates that genes involved in NK cell-DC interactions are upregulated in MLNs of mice infected with T1L but not T3D-RV (data not shown). Genes involved in NK cell-mediated cytotoxicity also are upregulated in MLNs during infection with T1L or norovirus strain CW3, both of which trigger inflammatory T cell responses to dietary antigen (15), suggesting that a common pathway induced by enteric viruses contributes to the onset of CeD. We aimed to elucidate the function of NK cells in reovirus infection and determine whether NK cells contribute to intestinal inflammatory responses that mediate LOT.

NK cells respond to cytokines, such as type I IFN, and release others, such as type II IFN (27–29). In the context of LOT, type II IFN can induce inflammatory DC activation and subsequent Th1 responses (28, 30–32). Type I IFN is required for T1L-induced dietary antigen-specific Treg suppression (14). However, how type I IFN leads to immune responses required for LOT is unknown. We found that NK cells both respond to type I IFN and regulate ISG expression in the MLNs following T1L infection. Type I IFN facilitates the infiltration and activation of NK cells in the MLNs and is required for production of type II IFN by NK cells during T1L infection (Figure 4). However, it is unclear whether the effects of type I IFN on NK cells are direct or indirect, as numerous signaling pathways, such as those induced by IL-15, are affected by type I IFN. Since IL-12 is dispensable for type II IFN production by NK cells during T1L infection (Supplemental Figure 2C). We conclude that the induction of type II IFN depends on type I IFN and does not rely on IL-12R/STAT4 signaling. The mechanism may be similar to type II IFN production by NK cells in the context of infection with other viruses, such as adenovirus, lymphocytic choriomeningitis virus (LCMV), and vaccinia virus (50–54), whereupon type I IFN directly induces the release of type II IFN by NK cells in the presence of additional stimuli. This signaling can be modulated by TLR/MyD88–induced STAT3 activation (55) or other cytokines such as IL-15 or IL-18 produced by DCs (56, 57), which is an area of research we will pursue in the future. We also observed a significant decrease in ISG expression in the MLNs following NK depletion (Figure 5). Therefore, NK cells contribute to type I IFN responses that are known to suppress dietary antigen-specific Tregs, which may explain the restoration of Treg priming following NK cell depletion during T1L infection (Figure 8).

Type II IFN can prime and activate DCs and Th1 cells. We discovered that NK cells produce type II IFN at early times following infection with T1L. CD103^+^CD11b^–^ migratory DCs in the intestine present peptides derived from dietary OVA to induce OVA-specific T cell responses (15). The DCs required for T1L-induced LOT express activation markers CD86 and CD8α and produce IL-12 (14). Following NK cell depletion, we observed significant decreases in both the number and percentage of CD103^+^CD11b^–^ DCs producing IL-12 and the number that express CD86 and CD8α (Figure 6, B and C), which induces Th1 T cell responses. Therefore, it is possible that type II IFN produced by NK cells contributes to CD103^+^CD11b^–^ DC inflammatory activation. However, administration of recombinant type II IFN in our OT mouse model was not sufficient to induce inflammatory Th1 responses specific to dietary antigen (data not shown). Further studies and mouse models would have to be established to deplete type II IFN specifically in NK cells to answer this question directly. However, we hypothesize that there are specific pathogen-host interactions that occur in addition to proinflammatory cytokine release to induce LOT, such as elaboration of pathogen-associated molecular pattern (PAMP) or damage-associated molecular pattern (DAMP) signaling.

Abrogation of immunological tolerance to dietary antigen is defined as the induction of inflammatory Th1 T cell responses and suppression of Treg induction (14, 15). We used an adoptive OT-II T cell transfer mouse model to investigate development of OVA-specific T cell responses following OVA administration and T1L infection. We found that NK cells both require and contribute to type I IFN responses in the MLNs (Figures 4 and 5), thereby suggesting a role for NK cells in T1L-induced LOT. Concordantly, we found that NK-cell depletion significantly blunts inflammatory OVA-specific Th1 T cell responses following T1L infection (Figure 8C). It is possible that type II IFN produced by NK cells contributes to Th1 T cell differentiation in the MLNs. Furthermore, Treg suppression, which requires type I IFN, was ameliorated during T1L infection in the absence of NK cells (Figure 8B). This finding suggests that NK cells contribute to T1L blockade of Treg expansion to dietary antigen, likely due to the significant decrease in type I IFN following NK cell depletion. Transcript levels of ISGs induced by type I IFNs in the MLNs are significantly decreased following NK cell depletion (Figure 5D). However, infectious virus is not detected in NK cells (Figure 3A) and, therefore, NK cells are not likely to be the primary producers of type I IFN during reovirus infection. However, infectious virus was detected in the total CD45^+^ immune cell population (Figure 3A), raising the possibility that NK cells recruit other innate immune cells that can be infected by T1L.

NK cells identify virus-infected cells using a “missing-self” mechanism, in which NK cells detect the absence of MHC class I expression on virus-infected cells, which is a common immune evasion strategy used by many viruses to avoid cytotoxic CD8^+^ T cell attack (58, 59). Failure to detect MHC class I molecules leads NK cells to kill virus-infected cells, thus aiding in viral clearance (35, 59, 60). NK cell depletion did not appear to diminish clearance of reovirus, despite the activation and elaboration of inflammatory cytokines following T1L infection. This finding suggests that either NK cells do not function in the detection and clearance of T1L-infected cells or that T1L avoids clearance by NK cells as an immune evasion strategy, analogous to adenovirus, hepatitis B virus, and herpesviruses such as human cytomegalovirus (61, 62). We found that NK cells contribute to host CD4 Th1 T cell responses early following infection (Figure 7) and dietary antigen-specific CD4 Th1 and Treg responses. Functions of NK cells in reovirus infection may be applicable to other enteric viruses. For example, norovirus CW3, which causes LOT to dietary antigen (15), may similarly induce NK-mediated inflammatory responses in the MLNs. Shared mechanisms of immune disruption by other enteric pathogens associated with virus-induced LOT could yield therapeutic targets for CeD prevention.

Our findings demonstrate that NK cells contribute to induction of dietary antigen-specific Th1 and suppression of Treg responses, but the effects are not complete. Other immune cells may synergize with NK cells to mediate DC and T cell responses associated with LOT and, therefore, depletion of NK cells is insufficient to fully restore tolerogenic responses to dietary antigen. We assessed innate immune cell profiles that differ between T1L and T3D-RV infection using t-distributed stochastic neighbor embedding analysis, and we identified non-NK cell clusters that vary between the 2 strains, including macrophages and inflammatory monocytes (data not shown). Characterization of these immune cell populations during reovirus intestinal infection may yield additional components of the LOT mechanism.

The identification of innate immune determinants of reovirus-induced LOT enhances our understanding of reovirus pathogenesis and the role of innate immune cells during intestinal infection, the requirement of these innate immune cells in reovirus-induced LOT, and, more broadly, requirements of a virus-induced trigger of an autoimmune disease. In turn, this information may advance new treatments for CeD, such as targeting innate immune cell populations or cytokines, and provide additional rationale for development of preventative vaccines.

## Methods

### Mice.

Mice used for these experiments were maintained on a C57BL/6 background. C57BL/6 (WT) and *IFNAR*^–/–^ (B6.129S2-Ifnar1tm1Agt/Mmjax) were purchased from Jackson Laboratories. *RAG*^–/–^ *OT-II*^+/–^ *CD45.1*^+/+^ mice were provided by Peter Savage. Mice were maintained in specific pathogen-free (SPF) environments at the University of Pittsburgh and University of Chicago and used for experiments at 6–8 weeks of age. *IFNAR^–/–^* were housed exclusively at the University of Pittsburgh. *RAG^–/–^ OT-II^+/–^ CD45.1^+/+^* were housed exclusively at the University of Chicago. Intestinal microbiota was controlled in WT and *IFNAR*^–/–^ mice by administering only irradiated food and using a water bottle after being weened. Fecal contents or cages were swapped a minimum of 2 times prior to infection. Animal husbandry and experimental procedures were conducted in accordance with Public Health Service policy and approved by the University of Pittsburgh’s and the University of Chicago’s Institutional Animal Care and Use Committees.

### Cell lines and viruses.

Spinner-adapted murine L cells were maintained in either suspension or monolayer cultures in Joklik’s modified Spinner MEM (SMEM) (Lonza) supplemented to contain 5% FBS (Thermo Fisher Scientific), 2 mM of L-glutamine, 100 U/mL of penicillin, 100 μg/mL of streptomycin (Thermo Fisher Scientific), and 25 ng/mL of amphotericin B (MilliporeSigma). Baby hamster kidney (BHK) cells that constitutively express the bacteriophage T7 RNA polymerase (BHK-T7 cells) were maintained in DMEM (Thermo Fisher Scientific) supplemented to contain 5% FBS, 2 mM of L-glutamine, 1 mg/mL of Geneticin (Thermo Fisher Scientific), and nonessential amino acids (MilliporeSigma).

Recombinant reoviruses were recovered using plasmid-based reverse genetics (63, 64). Strain T1L was recovered using plasmid-based rescue from cloned T1L cDNAs. Reassortant strain T3D-RV was recovered following transfection of BHK-T7 cells with plasmid constructs encoding the S1 and L2 gene segments from strain T1L and the remaining 8 gene segments from T3D. After 3–5 days of incubation, cells were frozen and thawed 3 times, and the virus was isolated by plaque purification using monolayers of L cells. Purified reovirus virions were purified from second- or third-passage L cell lysate stocks. Viral particles were extracted from infected cell lysates using Vertrel XF (DuPont), layered onto 1.2–1.4 g/cm3 CsCl gradients, and centrifuged at 62,000 x 3 *g* for 16 hours. Bands corresponding to virions (1.36 g/cm3) were collected and dialyzed in virion storage buffer (150 mM NaCl, 15 mM MgCl2, and 10 mM Tris-HCl [pH 7.4]). Viral titer was determined by plaque assay using L cells. Purified viral particles were electrophoresed in SDS-polyacrylamide gels, which were stained with ethidium bromide to visualize viral gene segments.

### Infection of mice.

Mice were PO inoculated with either 1 × 10^8^ or 1 × 10^10^ PFU of purified reovirus diluted in 100 μL of PBS using a 22-gauge round-tipped needle (Cadence Science). Titers of virus in the inoculum were determined to confirm the number of infectious particles in the administered dose. Viral loads in intestinal tissue or fecal contents were determined at various intervals after inoculation. Mice were euthanized, and a 3 cm ileum section for each mouse along with fecal contents were harvested into 1 mL of PBS and stored at −20°C prior to assay. Samples were thawed (25°C), homogenized using a TissueLyser LT (Qiagen) for 8 minutes using 5 mm stainless steel beads (Qiagen), frozen (−20°C), and homogenized again for 5 minutes prior to dilution for plaque assay. Viral loads in GALT including PPs and MLNs were determined using single cell suspensions obtained after tissue processing by flow cytometry. Suspensions were frozen at −20°C and thawed twice prior to dilution for plaque assay. Viral titers in organ homogenates were determined as the number of PFU per mL of tissue homogenate.

### Antibodies and flow cytometry.

The following fluorophore-conjugated antibodies were purchased from eBioscience: Tbet (4B10), Foxp3 (FJK-16s), Rat IgG2a isotype control, IL12/23p40 (C17.8), and F4/80 (BM8). The following antibodies were purchased from BD Biosciences: CD8a (53-6.7), IFN-γ (XMG1.2), CD69 (H1.2F3), CD11c (N418), and Fc Block (2.4G2). The following antibodies were purchased from BioLegend: GM-CSF (MP1-22E9), ki67 (16A8), Ly6C (HK1.4), CD86 (GL-1), CD45.2 (clone 104), CD49b (DX5), Ly6G (1A8), CD19 (1D3), F4/80 (BM8), CD64 (X54-5/7.1, NK1.1 (PK136), CD103 (2E7), MHCII (M5/114), and TCRb (H57-597). Aqua LIVE/DEAD Fixable Aqua Dead Cell Stain Kits were purchased from BioLegend. Cells were permeabilized using the Foxp3 Fixation/Permeabilization Kit (eBioscience) for transcription factor staining or Cytofix/Cytoperm (BD Biosciences) for cytokine staining. Flow cytometry was conducted using either a Fortessa (BD Biosciences) or Aurora (Cytek) flow cytometer. Flow cytometry data were analyzed using FlowJo software (Treestar), and flow cytometry Ab panels were designed using Fluorofinder. Sorting experiments were conducted using either an Aria II 3 laser (BD Biosciences) or an Aria Fusion (BD Biosciences).

### Isolation of PPs and MLNs.

PPs and MLNs were removed, treated with collagenase VIII (MilliporeSigma) at 37°C for 30 minutes, quenched with 10 μL of 0.5M EDTA, pH 8.0 (Corning), and mechanically disrupted by passage through a 100 μm cell strainer (14). Cell counts were determined using a hemocytometer following staining of an aliquot of a single cell suspension with 0.4% trypan blue (Thermo Fisher Scientific).

### Assessing NK cell cytokine production.

MLNs were resected and processed for flow cytometry. Approximately 2.5 × 10^6^ cells/sample were stimulated with 1 μg/mL leukocyte activation cocktail with GolgiPlug (BD Bioscience) at 37°C for 4 hours and stained for intracellular type II IFN following fixation and permeabilization.

### Immune cell and cytokine depletion.

To deplete NK cells, mice were i.p. injected with 300 μg of anti-NK1.1 depleting Ab (PK136, Bio X Cell) or InVivoPlus mouse IgG2a isotype control (Bio X Cell) 1 day prior to and following PO inoculation of reovirus. NK cell depletion was assessed using flow cytometry of single-cell suspensions of blood, spleen, and MLNs at 2 dpi and MLNs at 3 dpi following staining with the NK cell-specific markers NK1.1 and DX5 following manufacturer’s recommendations. To deplete IL-12, mice were i.p. injected with 750 μg of anti–IL-12p40 depleting Ab (C17.8, Bio X Cell) or InVivoPlus mouse IgG2a isotype control (Bio X Cell) following previously established protocols (65–67).

### In vivo T cell conversion assays.

T cell conversion in vivo was assessed by purifying CD4^+^ T cells from the spleen and lymph nodes of *RAG^–/–^ OT-II^+/–^ CD45.1*^+/+^ mice using a CD4^+^ T cell isolation kit (Miltenyi Biotec) or flow cytometry with a FACS Aria Fusion. Approximately 1 × 10^5^ cells were transferred retro-orbitally into congenic naive C57BL/6 mice. A day after transfer, mice were PO inoculated with 1 × 10^8^ PFU of reovirus T1L or PBS as a control and fed an OVA-containing diet (10 mg/kg; TD 130362, Harlan Envigo) for 3 days. Mice were euthanized at 3 dpi, and intranuclear levels of Foxp3, T-bet, or cytokine IFN-γ in transferred CD45.1^+^ and recipient T cells from MLN were determined by flow cytometry. Cells were incubated in the presence of 50 ng/mL PMA, 500 ng/mL ionomycin (MilliporeSigma), and 1.3 μL/mL Golgi Stop (BD Biosciences) at 37°C for 2 hours prior to flow cytometry.

### Reverse transcription PCR (RT-PCR).

RNA was prepared using the RNeasy Mini Kit (Qiagen). Expression analysis was conducted in duplicate using real-time PCR. Expression levels were quantified and normalized to GAPDH expression (Mm99999915_g1). RT-PCR was conducted using ready-to-use primer and probe sets (TaqMan Gene Expression Assays). Genes assessed were IFN-γ (Mm01168134_m1), MX1 (Mm00487796_m1), IFIT1 (Mm00515153_m1), and IRF1 (Mm01288580_m1).

### Statistics.

Experiments were conducted in duplicate or triplicate and repeated at least twice. Representative results of single experiments are shown in the figures as indicated. Mean values were compared using the Mann-Whitney test, 1-way ANOVA with multiple comparisons, or 2-way ANOVA. ANOVA was followed by Tukey’s post hoc test. The statistical test used and *P* values are indicated in each figure legend. *P* values of < 0.05 were considered statistically significant.

### Study approval.

All animal work reported here conforms to Public Health Service policy and was approved by the Institutional Animal Care and Use Committees at the University of Pittsburgh and University of Chicago.

## Author contributions

PHB, EK, BJ, and TSD conceptualized the study. PHB, EK, KLF, KU, and LMS conducted the investigation. RH, EK, and BJ provided resources. PHB and TSD wrote the original draft, and PHB, EK, KLF, GMT, BJ, and TSD reviewed and edited the manuscript. GMT, RH, BJ, and TSD supervised the project. PHB, BJ, and TSD acquired funding.

## Supplementary Material

Supplemental data

## Version 1 08/22/2022

Electronic publication
