# Supplementary material for: NK cells contribute to reovirus-induced IFN responses and loss of tolerance to dietary antigen
Source: JCI Insight. 2022 Aug 22;7(16):e159823. doi: 10.1172/jci.insight.159823 (PMC9462493; doi:10.1172/jci.insight.159823)
Supplement: Supplemental data [file jciinsight-7-159823-s168.pdf]

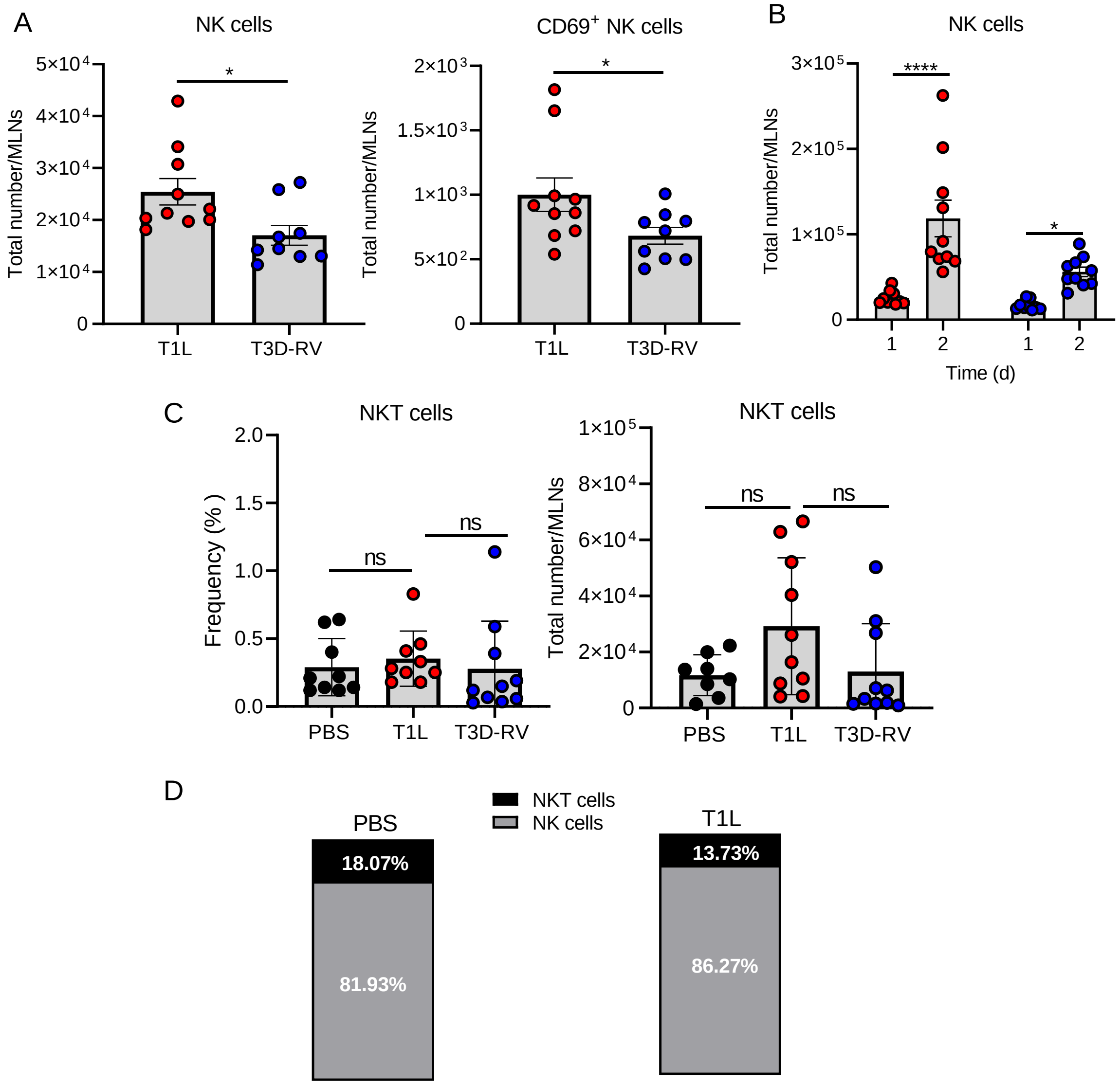

Supplemental Figure 1. Natural killer cells are the primary NK1.1+ population in the MLNs following T1L infection. WT mice were inoculated perorally with 10^8 PFU of T1L or T3D-RV or PBS as a control. **(A, B)** At 1 or 2 dpi, MLNs were resected and processed for flow cytometry. Single-cell suspensions were stained with a comprehensive antibody panel and analyzed by flow cytometry (n = 8-11). **(A)** Total cell count of NK cells ($CD45^+$ $TCR\beta^-$ $NK1.1^+$) or CD69-expressing NK cells in MLNs at 1 dpi. **(B)** Total cell count of NK cells ($CD45^+$ $TCR\beta^-$ $NK1.1^+$) or total cell count of CD69-expressing NK cells ($CD45^+$ $TCR\beta^-$ $NK1.1^+$) in MLNs at 1 or 2 dpi. **(C, D)** At 2 dpi, MLNs were resected and processed for flow cytometry (n = 9-10). **(C)** Percent frequency and total cells per MLNs of NK T cells ($CD45^+$ $TCR\beta^+$ $NK1.1^+$) **(D)** Percentage of NK or NK T cells of total $NK1.1^+$ $CD45^+$ cells from MLNs. Results are presented as mean values. Error bars

indicate standard errors of the mean (SEM). Statistical significance was calculated using Student's t test **(A)** or a one-way ANOVA with Tukey's multiple comparisons test **(B, C)**. *, $P < 0.05$; ****, $P < 0.0001$.

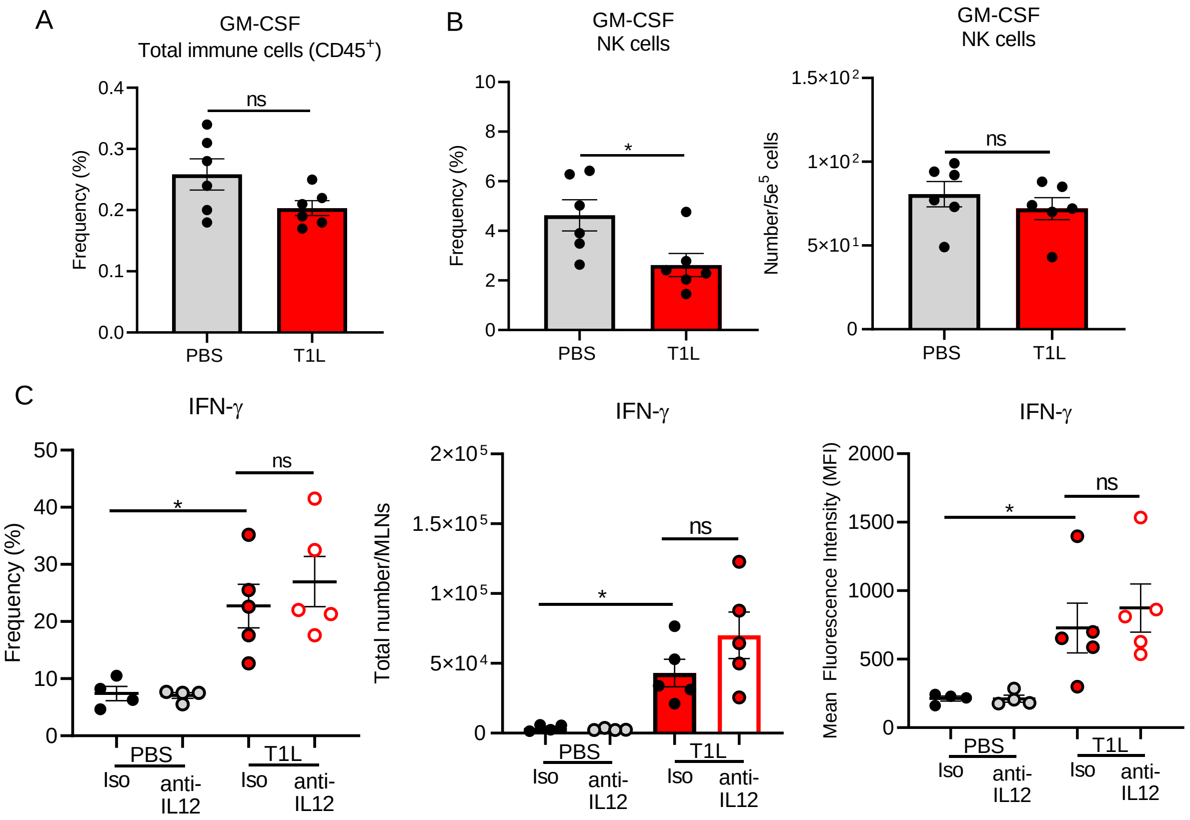

Supplemental Figure 2. NK cells do not produce GM-CSF following T1L infection and IL-12 is not required for type II IFN production by NK cells. (A, B) WT mice were inoculated perorally with 10^8 PFU of T1L or PBS as a control. At 2 dpi, MLNs were resected and processed for flow cytometry. MLN cell suspensions (5×10^6 cells) were stimmed with PMA and ionomycin at 37ºC for 4 hours, and cells were assessed for GM-CSF production by intracellular cytokine staining. **(A)** Expression of GM-CSF in total immune cells ($CD45^+$) or **(B)** percent frequency or total number of GM-CSF in NK cells ($CD45^+$ $TCR\beta^-$ $NK1.1^+$). (n= 6) **(C)** WT mice were intraperitoneally injected with either isotype control IgG2a antibody or anti-Il-12p40 (C17.8) antibody one day prior to and one day following PO inoculation with $10^{\mathbf{8}}$ PFU of T1L or PBS as a control. At 2 dpi, MLNs were resected and processed for flow cytometry. MLN cell suspensions (5×10^6 cells) were stimmed with PMA and ionomycin at 37ºC for 4 hours, and cells were assessed for IFN-γ production by intracellular cytokine staining. Total number, percentage, and MFI of NK cells ($CD45^+$ $TCR\beta^-$ $NK1.1^+$) that express intracellular IFN-γ are plotted in **(C)** (n=4-5). Results are presented as mean values. Error bars indicate standard errors of the mean (SEM). Statistical significance was calculated using

Student's t test **(A, B)** or one-way ANOVA with Tukey's multiple comparisons test **(C)**. *, $P < 0.05$; **, $P < 0.01$; ***, $P < 0.001$; ****, $P < 0.0001$.

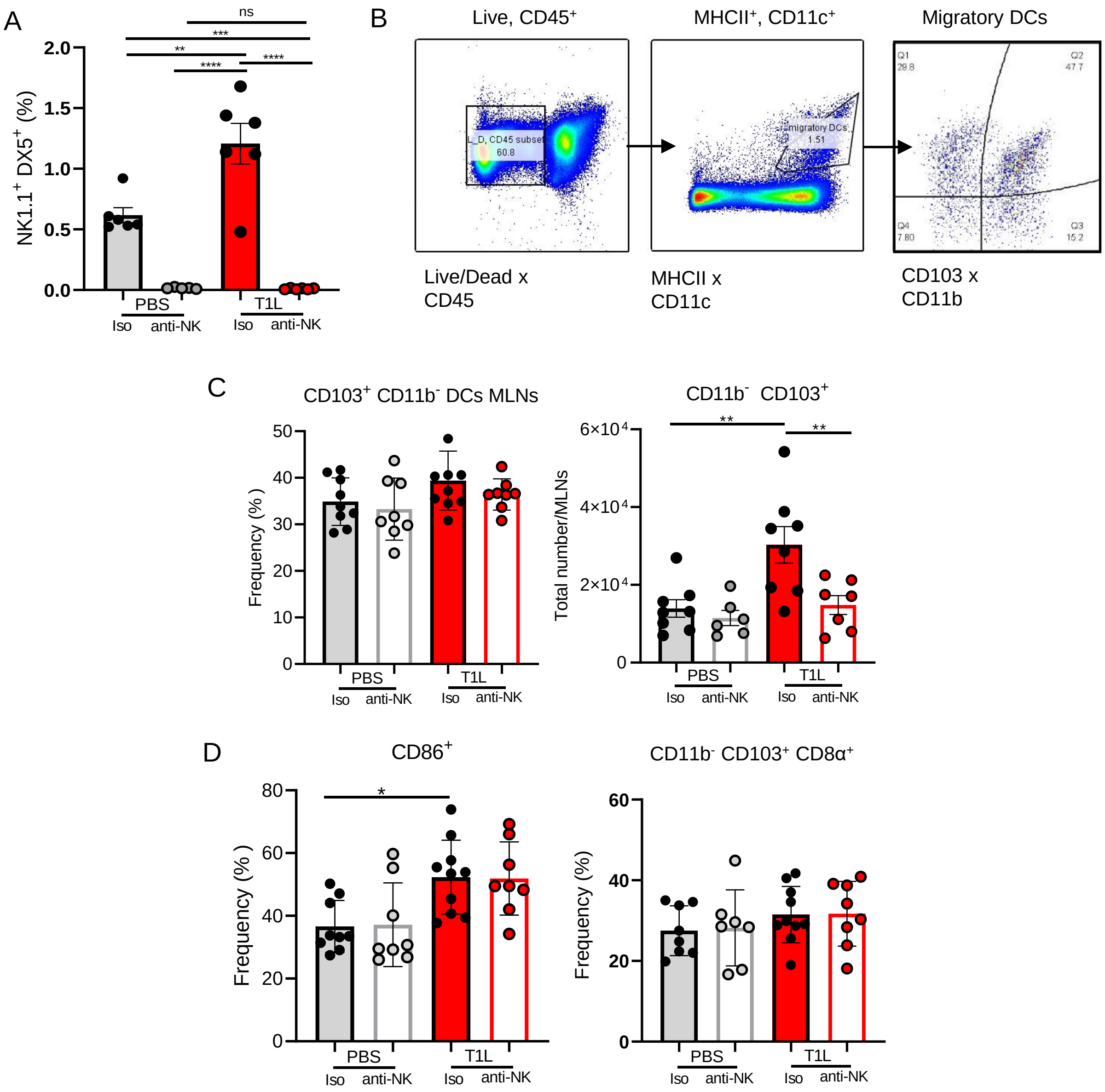

Supplemental Figure 3. Characterization of migratory DCs during T1L infection following NK cell depletion. WT mice were intraperitoneally injected with either isotype control IgG2a antibody or anti-NK1.1 antibody (PK136) one day prior to and one day following PO inoculation with 10^{8} PFU of T1L or PBS as a control. At 2 dpi, MLNs were resected and processed for flow cytometry. Single-cell suspensions were incubated with brefeldin A in the presence of Golgi Plug at 37ºC for 6 hours (n = 8-10). **(A)** Validation of NK cell depletion in MLNs. **(B)** Example flow gating strategy for $CD103^{+}$ $CD11b^{-}$ migratory DCs in the MLNs. **(C)** Frequency and total number of migratory tolerogenic DCs ($CD11c^{int}$ $MHCII^{+}$ $CD103^{+}$ $CD11b^{-}$) in the MLNs (n=6-8). **(D)** Frequency of $CD103^{+}$ $CD11b^{-}$ that express CD8α or CD86 (n=6-8). Results are

presented as mean values. Error bars indicate standard errors of the mean (SEM). Statistical significance was calculated using one-way ANOVA with Tukey's multiple comparisons test. *, $P < 0.05$; **, $P < 0.01$; ***, $P < 0.001$; ****, $P < 0.0001$.
